# The patronage of religious tourism seen from its motivations that predict satisfaction and loyalty: The Virgin of Chaguaya in Bolivia

**DOI:** 10.1371/journal.pone.0307664

**Published:** 2024-08-13

**Authors:** Mauricio Carvache-Franco, Jose Loaiza-Torres, Orly Carvache-Franco, José Eduardo Fernández-Cruz, Wilmer Carvache-Franco

**Affiliations:** 1 Universidad Bolivariana del Ecuador, Durán, Ecuador; 2 Centro de Investigación en Ciencias Sociales y Empresariales (CICSE), Universidad Católica Boliviana San Pablo, Tarija, Bolivia; 3 Universidad Católica de Santiago de Guayaquil, Guayaquil, Ecuador; 4 Centro de Investigación en Ciencias Sociales y Empresariales (CICSE), Universidad Católica Boliviana San Pablo, Tarija, Bolivia; 5 Escuela Superior Politécnica del Litoral, ESPOL, Facultad de Ciencias Sociales y Humanísticas, Guayaquil, Ecuador; SEGi University Kota Damansara, MALAYSIA

## Abstract

This study focused on pilgrimages as part of religious tourism and aimed to achieve the following objectives: identify the main motivational factors of religious tourism focused on pilgrimages; analyze the motivational dimensions that predict satisfaction in religious tourism focused on pilgrimages; analyze the motivational dimensions that predict loyalty in religious tourism focused on pilgrimages. The study was conducted during the pilgrimage to the Virgin of Chaguaya in Bolivia. The sample consisted of 384 tourists who were surveyed on-site. The statistical techniques used included factor analysis and multiple regression. The results revealed four motivational dimensions: Tourism and Escape, Religious Experience, Belief Experience, and Shopping. Additionally, specific motivations that influence the satisfaction and loyalty of attendees at religious events such as pilgrimages have been identified, among them the "Religious Experience" and the "Belief Experience" motivational dimensions. The findings will contribute to planning and management guidelines for religious event administrators and provide information to academic literature.

## 1. Introduction

The study of religious tourism allows us to explore aspects such as motivations for demand, satisfaction and loyalty. These interconnected variables drive the continued development of research in this field of tourism. In this sense, religious tourism is a form of travel for religious purposes or with a religious orientation [1]. It also provides a meaningful experience for those seeking a deeper connection with their faith [2]. Religious tourism refers to an essentially subjective experience of spirituality [3] that analyzes the multifunctional nature of pilgrimage journeys [4]. Religious tourism offers unique opportunities [5] and, despite the secularization and popularization of religions, continues to attract a large number of tourists [6].

Thus, to understand religious tourism it is important to analyze the motivations of those attending religious events. Motivations refer to the individual characteristics of each tourist related to the journey [7]. Tourist motivation enables an understanding of tourist destination choices, behaviors, and travel needs and expectations [8]. Consequently, motivation in religious tourism would be the driving force to travel to a sacred place [9–11]. In this sense, religious tourists may have various travel motivations, such as personal fulfillment, the desire to participate in religious rituals or to offer prayers and vows [4].

On the other hand, tourist satisfaction is a psychological state that tourists experience regarding a destination’s service or product [12]. Also, indicated that a positive perception of tourism leads to higher satisfaction, thus fostering loyalty [13]. Thus, loyalty is precisely conceived as the repetition of visits to attend the same event [14]. According to Lu et al. [15] the sense of awe experienced when in contact with environments such as religious ones can impact tourists’ loyalty and, therefore, influence their behavior and decisions. Moreover, Al- Msallam [16] stated that the positive impact of tourists’ emotions regarding satisfaction and loyalty to the destination leads to a tendency to revisit the destination; in this regard, loyalty is a crucial determinant in the intention to revisit [13]. In this sense, tourists’ loyalty increases with tourist satisfaction originating from their motivations [17].

This work will analyze the festival of the Virgin of Chaguaya in Tarija, Bolivia, a reference point for religious tourism in the region. This Catholic celebration is one of the country’s biggest, attracting national and international tourists. Its origin dates back to 1750, with the appearance of the Virgin to several peasants in the area, who, after this vision, would have taken the image of the Virgin to their home; sometime later, a chapel was built in her honor. She is known as the "Mamita de Chaguaya," and the celebrations in her honor occur on the first Sunday of August and extend until September 15th each year.

In this order of ideas, demand studies in religious tourism are very important because they serve to establish management plans for the administrators of these places where these events take place. Improving the activities of religious events will provide attendees with a longer stay together with their family and friends. Those attending religious events, if they find facilities and products according to their needs, will return at future events. One of the main religious events are pilgrimages where people travel to a certain sanctuary or sacred place with important religious connotations. In this sense, the academic literature is diverse in relation to the motivations found by different authors in various religious pilgrimages. Therefore, establishing the motivations for religious tourism is crucial for the management of destinations. It is also very important to find the relationship between the motivations with the satisfaction and loyalty of those attending religious tourism focused on pilgrimages. Therefore, this study addresses the following objectives in the Virgin of Chaguaya Pilgrimage in Tarija in Bolivia: i) identify the main motivational factors of religious tourism focused on pilgrimages, (ii) analyze the motivational dimensions that predict satisfaction in religious tourism focused on pilgrimages, (iii) analyze the motivational dimensions that predict loyalty in religious tourism focused on pilgrimages.

## 2. Literature review

### 2.1. Motivations for demand in religious tourism

Religious tourism focuses on pilgrims and tourists’ visits to places of religious significance [9, 18]. It presents different motivations, such as religion, religiosity, culture, and the desire for healing [19]. Currently, it attracts a high number of tourists and visitors despite the secularization and popularization of religions worldwide [6], as it could provide a deep connection with faith by combining the human and the divine [20–22]. On the other hand, tourist motivations are the specific desires of tourists that lead them to travel or choose a destination [8]. These would originate from the internal motives of the trip and the actions that tourists carry out as part of structured and unstructured means [21, 23].

In this context, several contemporary studies on motivations in religious tourism stand out, such as Lois–González & Santos [24], who identified religious, cultural, sports, and shopping motivations. In a study conducted on the island of Lesbos in Greece, the main motivations of visitors to religious sites, including churches and monasteries, were analyzed. Motivations related to pilgrimages and spiritualism were present, as well as cultural and secular dimensions [25].

Similarly, a study focused on tourists and traditionally religious sites in Hungary discovered that the need for personal fulfillment, destination culture, the desire to be part of a religious ritual, and emotional sense were motivations for visitors to these sites [4]. Likewise, the Church of St. Nicholas in Antalya, Turkey, served as the basis for the study by Bideci & Albayrak [26], which found religious and spiritual motivations in Russian and German tourists who visited this site. These motivations vary depending on the nature of the site and whether or not they seek the divine, thus identifying the following factors: religiosity, sense of belonging, and spiritual stimulation. Similar factors were analyzed in the work of Pillai et al. [27], which identified five types of motivations in tourists who visited the relics of St. Francis Xavier in Goa, India: social exploration, religion and belief in the experience, escape, and shopping. Quoting a study on the Muslim religion that analyzed the pilgrimage called Umrah, which is directed to the city of Mecca in Saudi Arabia, five classes of motivations were found: gratitude, spiritual integration, seeking opportunities for spiritual growth, spiritual interest, and a sense of peace [28]. Another study based on the motivations of tourists who visited the Sanctuary of Lourdes in France identified the search for faith, purpose, and healing as the most prominent [20].

Similarly, a study identified religious, spiritual, and cultural motivations as frequent in religious tourists who visited the Romanian monastery of Prislop [29]. Likewise, researchers Piramanayagam et al. [30] examined travel motivation factors in pilgrims to Bodhgaya, a Buddhist site in northern India, highlighting motivations such as religious beliefs, culture, and attractions. One study analyzed the motivations of tourists who visited Roman Catholic shrines in Poland. This work identified five categories: religious motivations, tourist motivations, recreation, social and family motivations, and commercial and shopping motivations [31].

Several of these motivational factors were found in the study by Hassan et al. [32], which focused on Muslim pilgrims who visited the city of Mecca in Saudi Arabia. This work expanded the literature on motivations in recent years by discovering new motivational dimensions such as social and the purchase of religious items. Another study on the sanctuary mentioned above, Lourdes, revealed motivations such as gratitude to God, complaints, and requests for help as those that stood out widely in their research based on the impact that the COVID-19 pandemic had on religious tourism in Lourdes [33]. Finally, a study by Kayal [34] on religious sites in Saudi Arabia showed that tourists’ intentions are related to their needs and self-esteem, leading the author to identify six types of motivations: spiritual, cultural, and educational; social and communicative; health and well-being; adventure and exploration; and fulfillment of religious obligations. The literature analyzed to frame this work indicated different perspectives on motivations. These, in turn, indicated the particularities of religious sites and their specific manifestations, such as pilgrimage; likewise, it was possible to understand tourists’ preferences regarding destination choice briefly and to specify a first research question. RQ1. What are the main motivations found in religious tourism focused on pilgrimages?

### 2.2. Relationship between motivations of religious tourism and satisfaction

Contemporary literature in this field has shifted from examining travel motives and tourist satisfaction to investigating the influence of travel experiences and their effect on future behavior [6, 19]. In religious tourism, satisfaction is related to destination motivations [35] and to the mediating effects of awe on tourists [15]. Researchers have indicated that satisfaction is the feeling of awe that positively impacts tourist loyalty and is related to positive emotions such as well-being and joy [36].

In this line, a study by Bond et al. [37] examined three Christian religious sites in England, including Canterbury Cathedral, the Shrine of Our Lady of Walsingham, and the Christian pilgrimage festival at Glastonbury Abbey. The aim was to understand the tourist experience and their satisfaction level. The findings revealed that tourists seek different experiences and obtain spiritual and cognitive benefits influencing their satisfaction. For another author, tourist satisfaction is a direct determinant of loyalty. This study focused on Islamic religious tourism and found several motivational factors for tourist satisfaction, including the functional, emotional, Islamic, price, and quality dimensions, which could influence Muslim tourists to choose a tourism service and foster their loyalty in the future [13]. Similarly, Kreiner et al. [38] emphasized that religious, geographical, and sociological aspects would be necessary within religious tourism after studying a conflict within the construction of the Baha’i World Center tourism center in Haifa (Israel).

Battour et al. [39] examined the relationship between tourist motivations and the satisfaction of Muslim tourists visiting Malaysia. The researchers emphasized how religion moderates this relationship, considered positive due to the religious attributes that could be complementary factors in tourists’ satisfaction; among them, the following stood out: the availability of halal food, the ease of finding mosques and places of worship, and security. Regarding religious tourism, Verma and Sarangi [14] analyzed the context of the Kumbh Mela, a massive pilgrimage of Hindu tourists held every 12 years in India. The study identified several motivations among tourists, such as religious faith, the pursuit of salvation, and the opportunity to experience a sense of community. It was observed that motivation attributes, service quality, and security influenced the pilgrims’ satisfaction.

An additional study based on the Pilgrimage to Santiago de Compostela identified several motivations in tourists, including the pursuit of holiness, emotional change, personal reflection, the desire to return to the place, adventure, satisfaction, physical training, and transformative experience. With this, it was demonstrated that in this pilgrimage, each tourist would experience a particular religious connection, although it may not always be a satisfying experience [40]. Likewise, research by Lin et al. [41] established a connection between satisfaction in religious tourism and personal well-being, which was especially relevant in risk contexts such as that caused by the COVID-19 pandemic. This study focused on the Baishatun Mazu temple in Taiwan and highlighted that religious activities could serve educational, recreational, stabilizing, and stress-relieving functions, which could increase tourist satisfaction. One of the latest works that analyzed satisfaction and its motivations in religious tourism discovered that motivational factors would be significant predictors of satisfaction. This work focused on one of the famous pilgrimages to Mecca in Saudi Arabia; according to the researchers, religious motivation was the determining factor influencing tourists’ satisfaction [32]. On the other hand, Shen et al. [42] studied various cities in Malaysia to analyze the perception of Islamic tourists and the impact of destinations on their satisfaction, revealing three dimensions of tourist satisfaction: satisfaction with offers and services, satisfaction with cultural authenticity, and satisfaction with the overall experience.

The literature analyzed in this section led the research toward a positive encounter between satisfaction qualities based on diverse motivations existing in the concrete experiences of tourists, pilgrims, and visitors. Understanding a close relationship between both variables could enhance new concepts and findings and consolidate more comprehensive research within religious tourism and its related areas. In this context, a second research question emerges RQ2. What are the main motivations that predict satisfaction in religious tourism focused on pilgrimages?

### 2.3. Relationship between the motivations of religious tourism and loyalty

Within religious tourism, loyalty is tourists’ intention to revisit a religious or sacred destination and their willingness to recommend it [13, 43]. Furthermore, it would be a significant moderator of place attachment [9] that would be linked to the results produced by satisfaction [34]. Therefore, Kim et al. [1] indicated that religious tourists usually show loyalty to the sacred destinations they visit. Likewise, Al-Msallam [16] indicated that loyalty would be a behavioral or non‐behavioral response derived from some generally positive emotion.

In that regard, Wang et al. [44] conducted a study based on the motivations of religious tourists visiting Buddhist mountains in China and discovered religious, cultural, and personal factors directly influencing tourist loyalty [44]. Meanwhile, researchers Tkaczynski & Arli [45] revealed that other theories on motivation and loyalty in religious tourism indicated that the study of motivations of a religious tourist is based on altruism and leadership in faith. Siregar et al. [46] analyzed Muslim destinations in Indonesia and focused their study on Muslim individuals, finding that the religious experience is crucial for tourist loyalty. They examined the main motivational factors present, including Muslim tourist attractions, the religious culture of tolerance in this area, and the pursuit of learning. Verma & Sarangi [14] analyzed the Kumbh Mela. According to the results of this study, the researchers concluded that tourists would be motivated by the services available in this pilgrimage. It would likely result in high satisfaction and potential loyalty to the destination. Huang et al. [47] focused on mainland China’s Four Great Buddhist Mountains, studying the primary factors and spiritual values that influence visitors, highlighting the pursuit of a balanced life, spiritual interaction, and the development of spiritual values. The authors indicated that these would achieve tourist satisfaction and improve their loyalty. Wu & Mursid [48] investigated the Umrah, an Islamic pilgrimage to Mecca (Saudi Arabia), and its impact on tourist loyalty. They found that interaction with the religious community and cultural experiences in Mecca are crucial for developing loyalty. After segmenting the data by similar sociodemographic aspects, they concluded that participation in this pilgrimage influences satisfaction and, in turn, travelers’ loyalty. A study focused on the Feast of the Church of Attur, also known as Jatre, among attendees of the Karkala festival (India). This manifestation showcases religious diversity (Christians, Hindus, and Muslims), so this work sought mediation between these. Additionally, the religious affiliation of participating tourists would moderate the relationship between attachment to the place and loyalty to the destination, with emotional experience mediating this relationship [49].

Similarly, in a new study based on the exact location, Hassan et al. [50] revealed that the religious factor would be the primary motivation for satisfaction and loyalty toward a sacred site. Researchers discovered four elements of pilgrim loyalty: influencing factors, impact of satisfaction, sense of belonging to the religious community, and management factors in religious tourism. A study conducted in India during the Kumbh Mela festival found that tourists show loyalty to places with religious events through their motivations, shared beliefs, emotional solidarity, and memorable religious experiences [51]. Another work on the Taoist temple of Zinan in Taiwan indicated that loyalty in religious tourism is the positive result of attachment to the place and tourist motivation, highlighting the indispensability and irreplaceability that emerged from their particular beliefs [42].

The research in this section suggests that tourists’ motivations in religious tourism are influenced by the visitor’s experiences and their concept of spirituality. Several authors agree that these motivations influence how tourists approach a religious destination, attributing value and meaning to the process experienced. This can influence the decision to return to the site or to recommend it to other tourists. With these aspects addressed, the third research question arises: RQ3. What motivational factors predict loyalty in religious tourism focused on pilgrimages?

## 3. Methodology

### 3.1. Study area

The study was conducted in the Plurinational State of Bolivia, Department of Tarija, where the pilgrimage to the Virgin of Chaguaya takes place, which is an invocation of the Virgin Mary that is venerated in the town of Chaguaya, located 67.5 km southwest of the city of Tarija. It is also known as the "The Assumption of Mary." Currently, the pilgrimage takes place between August 15th and September 15th of each year, and it has been widely spread that pilgrims attend both from the interior of Bolivia and from abroad, such as from Northern Argentina, traveling on foot between approximately 65 and 70 kilometers.

According to the legend, one day in 1750, a couple of shepherds were returning from their work and discussing the problematic situation due to that year’s drought; they talked about how rain would be the only salvation for the crops and animals. As night fell, the couple hurried along with their sheep and goats; suddenly, they spotted a glow not far away. Curious, they stopped to head towards the light; as they approached, the light turned multicolored, and the rays intertwined, giving an incredible sight; both were absorbed in contemplating this play of lights, which gradually gave way to a beautiful image of the Virgin Mary on the top of a "molle" tree, which is a typical tree of the area; they fell to their knees and kissed the ground exclaiming, "Holy Mary, blessed be God!" Moments later, when they raised their heads, the image was no longer there. The next day, at dawn, they went to the place of the apparition, and there was the image; they took it to their ranch, placing it in a place of preference. Once the neighbors heard about it, they went to the house to venerate the Blessed Virgin, but they were surprised to find that the image had disappeared; quickly, everyone went to the place of the apparition; indeed, the image lay under the leafy tree, enveloped in a ray of light; they knelt and prayed all night, and other locals joined them with torches and bonfires, accompanying the image; then they promised to build a chapel in that same place, so the next day the villagers built the first chapel where the image of the Virgin of Chaguaya was venerated [52].

In the 1980s, the current sanctuary was built and declared the Basilica of the Virgin of Chaguaya. The main objective of the pilgrims is to reach the feet of the Virgin to pray, ask for their intentions to be fulfilled, thank for the favors received, make themselves "stepped on," and renew their promise of faith, thus returning the following year to fulfill their promise. Within the traditions, specific steps can be grouped into seven [53]. 1. The promise consists of a personal commitment to go to Chaguaya to be with the Virgin to give thanks and ask for blessings; 2. The pilgrimage, a walking tour that lasts approximately 12 hours, is the execution of the promise and is considered a sacrificial offering, 3. The confession that cleanses, erases, purifies, and liberates, 4. The footprint that is a personal encounter with the Virgin, 5. Eucharist and communion that nourishes and comforts, 6. The road to Calvary is a gesture that the Virgin accompanies us at all times, mainly in moments of pain and suffering, 7. Gastronomic enjoyment with the typical food of the place, such as chirriadas, natural cane juice, peanut soup, spicy chicken, and others. Although the last step is not necessarily religious, it is part of the pilgrimage. After being nourished and receiving the blessing of the Mamita de Chaguaya, they return to their homes strengthened, promising to return for the following year.

On the other hand, one crucial step that can be added to the indicated ones is participating in the procession. This activity takes place immediately after the Eucharist around the courtyard of the Virgin’s temple. This activity shows how the faithful carry the image of the Virgin during the procession, thus demonstrating their devotion.

### 3.2. The survey, data collection, and analysis

The present study is part of the Project that was approved by the Research Dean of the ESPOL University and the Ethics Committee of the ESPOL Polytechnic University with code FCSH-14-2021. Informed consent was requested in writing and was part of the attached Questionnaire. Due to religious ethical aspects, the study was analyzed by the authors and a group of specialists to establish that the results are only technical and do not clearly involve aspects of religious faith.

The present study the data were measured through a quantitative study that used the questionnaire as a data collection instrument. This content three sections. For its design, several research works were first reviewed as sources of information available in religious tourism; during the questionnaire development phase, the research team selected previous works that would serve to design the data collection instrument. The first section contained sociodemographic aspects and consisted of 7 closed questions based on the study by Lee et al. [54]. The second part of the questionnaire comprised 22 items from the study, Pillai et al. [27], adapted to the main motivations previously discussed in this work. The motivation scale was measured using a 5-point Likert scale (1 not very important to 5 very important). The Cronbach’s Alpha coefficient of the final motivation scale reached a value of 0.904, indicating high internal consistency among the scale items. The third part of the questionnaire consisted of satisfaction and loyalty variables adapted from the study [55]. For this purpose, the satisfaction scale contained one item on a 5-point Likert scale (1 strongly disagree to 5 strongly agree). Additionally, the loyalty scale comprised three items measuring return, recommendation, and positive word-of-mouth and was also developed on a 5-point Likert scale (1 strongly disagree to 5 strongly agree). To validate the questionnaire, a group of experts analyzed and improved it, and then a pilot test was conducted with ten individuals to reduce limitations and possible biases and prevent questions from being biased and avoid problems in interpreting the options.

The sampling was carried out after the interviewers had been trained. The survey was applied when the people had already finished the tour from August 26 to August 27, 2022, completing the required number of surveys without any inconvenience, given the number of pilgrims carrying out the pilgrimage.

The research team used convenience sampling, which determined the willingness of pilgrims to participate in the questionnaire. The achieved sample size was 384, indicating a margin of error of +/- 5%, a confidence level of 95%, and a variation of 50% based on an infinite population to calculate the sample. Then, the surveys were entered for statistical analysis in the SPSS Version 26 program. Among the statistical techniques used was factor analysis, which was employed to reduce items into factors that improve the interpretation of values. A Varimax rotation method was used to arrange the factorial loads. The Kaiser method was also used to find the number of factors according to eigenvalues. The KMO (Kaiser-Meyer-Olkin) measure was another important indicator for validating the model. Bartlett’s sphericity test was used to determine the appropriateness of the factorial analysis model. Another technique utilized was multiple regression analysis, which found significant predictors influencing satisfaction variables, intentions to return, intentions to recommend, and intentions to speak positively about the religious pilgrimage.

## 4. Results

### 4.1. Sample profile

According to the analyzed sample, there was a higher percentage of men, 57.9%. The highest percentage was for those under 20 and between 20 and 29 years old. The majority were single (71.6%). 45% were university students. 11.3% were public employees and 11% were private employees. A higher percentage attended the pilgrimage with family (49.6%), and 39.9% attended with friends. A high percentage (41%) spent less than $50 on the pilgrimage. See Table 1.

**Table 1:** Sociodemographic variables.

Variables	Categories	N	%
Gender	Male	222	57.9
	Female	162	42.1
Age	Less than 20 years old	153	39.9
	20 to 29 years old	105	27.3
	30 to 39 years old	41	10.7
	40 to 49 years old	31	8
	50 to 59 years old	33	8.6
	More than 60 years old	21	5.4
Marital status	Sigle	275	71.6
	Married	88	22.8
	Other	22	5.6
Educational level	Primary	41	10.7
	Secondary	118	30.8
	University	173	45
	Postgradute / Master / Ph.D	51	13.4
Occupation	Student	220	57.4
	Researcher/Scientist	1	0.3
	Businessman	20	5.1
	Private Employee	42	11
	Public Employee	43	11.3
	Pensioner	9	2.4
	Unemployed	13	3.5
	Other	35	9.1
With whom did you attend the Pilgrimage of the Virgin of Chaguaya?	Alone	15	4
	With your family	190	49.6
	With friends	153	39.9
	With your partner	23	5.9
	Other	2	0.5
What is your daily expense on your visit to the Pilgrimage of the Virgin of Chaguaya?	Less than $50	159	41.3
	$50 - $99	101	26.3
	$100 - $149	86	22.5
	$150 - $199	17	4.3
	$200 - $249	11	2.9
	More than $250	10	2.7

### 4.2 Motivations in religious tourism

A Factor Analysis was used to reduce the items into factors that enhance the interpretation of the results. The Varimax rotation method arranged the factor loadings into high and low ones. The Kaiser method was also used to find the appropriate number of factors with eigenvalues greater than 1. The Cronbach’s Alpha of the factors ranged from 0.823 to 0.494, indicating a high correlation among the elements comprising each factor. The KMO was 0.902, a value close to 1, and Bartlett’s test of sphericity was significant, indicating the appropriateness of conducting the factor analysis. See Table 2.

**Table 2:** Motivations in religious tourism.

Variable	Tourism and Escape	Religious Experience	Belief Experience	Shopping
It is an opportunity to get to know Tarija	0.801			
To satisfy curiosity	0.787			
To escape from the routine	0.772			
To relieve boredom	0.767			
For tourism	0.751			
To relieve daily stress	0.731			
For vacation	0.684			
To join friends and family	0.678			
To share the experience with other believers	0.494			
Appreciate/experience the beauty of the sanctuary		0.705		
To seek peace		0.692		
To experience the mystery of religion		0.692		
To seek spiritual comfort		0.670		
To appreciate its architecture		0.670		
To attend the religious festival		0.558		
To experience a holy atmosphere			0.823	
For religious fulfillment			0.797	
To pay respect to the relics of the Virgin			0.648	
To fulfill a lifelong wish			0.621	
To redeem/free me from suffering			0.606	
To buy religious items				0.701
To buy local products				0.587
Cronbach’s alpha	0.898	0.82	0.827	0.735
Eigenvalues	7.422	3.785	1.311	1.046
% explained variance	33.735	17.206	5.960	4.756
% cumulative variance	33.735	50.941	56.901	61.657

According to the results in Table 2, the first dimension was related to tourism activities and the escape from daily routine and stress. For this reason, this factor was labeled as "Tourism and Escape." This factor accounted for 33.74% of the explained variance. On the other hand, the second dimension was named "Religious Experience" as it was related to the sanctuary, seeking peace, and spiritual comfort. This factor comprised 17.21% of the explained variance.

Meanwhile, the third dimension was associated with experiencing a wholesome atmosphere, religious fulfillment, and respect for the Virgin’s relics. Therefore, it was labeled as "Belief Experience." This factor explained 5.96% of the variance. In contrast, the fourth dimension was termed "Shopping," as it was related to purchasing religious articles and local products and answered our first research question: RQ1. What are the main motivations found in religious tourism focused on pilgrimages?

### 4.3 Motivations and satisfaction

A multiple regression is used to find the significant predictors for the dependent variable satisfaction. See Table 3.

**Table 3:** Motivations and satisfaction (significant predictors).

Variable	Beta (standardized)	t	Sig.	Tolerance
Tourism and Escape	-0.207	-4.623	0.000	1.000
Religious Experience	0.293	6.530	0.000	1.000
Belief Experience	0.363	8.091	0.000	1.000
Shopping	-0.028	-0.628	0.530	1.000
(Constant)		124.332	0.000	
F Test	32.466			
Sig.	0.000			
Durbin Watson	1.677			

According to the results in Table 3, the regression model was significant (p < 0.05), with a tolerance close to 1 (high tolerance), indicating the absence of multicollinearity. The Durbin-Watson statistic ranged between 1.5 and 2.5, indicating no autocorrelation in the residuals. The dimension "Belief Experience," with a beta intensity of 0.363, showed a significant positive correlation with satisfaction, which means that attendees (of the religious pilgrimage) with higher motivations for Belief Experience were more satisfied. "Religious Experience" was another dimension positively correlated with satisfaction, showing a beta of 0.293, indicating that attendees (of the religious pilgrimage) with a greater religious experience had high satisfaction. In contrast, the "Tourism and Escape" dimension negatively correlated with satisfaction. Thus, attendees with high motivations for Tourism and Escape had lower satisfaction with the event, which answers our second research question: RQ2. What are the main motivations that predict satisfaction in religious tourism focused on pilgrimages?

### 4.4 Motivations and intentions to attend the pilgrimage again

The multiple regression method was used to analyze the significant predictors in the variable Intentions to attend the pilgrimage again. See Table 4.

**Table 4:** Motivations and intentions to return (significant predictors).

Variable	Beta (standardized)	t	Sig.	Tolerance
Tourism and Escape	-0.068	-1.442	0.150	1.000
Religious Experience	0.292	6.213	0.000	1.000
Belief Experience	0.308	6.572	0.000	1.000
Shopping	-0.065	-1.392	0.165	1.000
(Constant)		100.158	0.000	
F Test	21.452			
Sig.	0.000			
Durbin Watson	1.945			

According to the results in Table 4, the model was significant and showed no multicollinearity or autocorrelation. Three dimensions were significant predictors (p < 0.05) for intentions to return to the pilgrimage. The "Belief Experience" dimension, with a beta intensity of 0.308, was the predictor that had a high positive correlation with intentions to return when attending the pilgrimage. Thus, attendees of the pilgrimage with high motivations for Belief Experience had higher levels of intention to return to the pilgrimage. Meanwhile, the "Religious Experience" dimension had a positive correlation (beta = 0.292) with intentions to return to the pilgrimage, indicating that attendees with high motivations for Religious Experience had high levels of intention to return to the pilgrimage.

### 4.5 The motivations and intentions of recommending the pilgrimage

The multiple regression method was used to analyze the significant predictors for the independent variable "Intentions to recommend the pilgrimage". See Table 5.

**Table 5:** Motivations and intentions to recommend (significant predictors).

Variable	Beta (standardized)	t	Sig.	Tolerance
Tourism and Escape	-0.065	-1.339	0.181	1.000
Religious Experience	0.284	5.894	0.000	1.000
Belief Experience	0.247	5.119	0.000	1.000
Shopping	0.009	0.186	0.853	1.000
(Constant)		94.631	0.000	
F Test	15.693			
Sig.	0.000			
Durbin Watson	1.803			

According to the results in Table 5, the model was significant and showed no multicollinearity or autocorrelation. Three dimensions were significant predictors (p < 0.05) for intentions to recommend the pilgrimage. The "Religious Experience," with a beta intensity of 0.284, positively correlated with intentions to recommend the pilgrimage. Therefore, attendees of the pilgrimage with high motivations for Religious Experience were the ones who had greater intentions to recommend the pilgrimage to others. On the other hand, the "Belief Experience" dimension (beta = 0.247) is positively correlated with intentions to recommend the pilgrimage. Thus, attendees of the pilgrimage with higher motivations for Belief Experience were the ones who had greater intentions to recommend the pilgrimage to others.

### 4.6 Motivations and saying positive things about the pilgrimage

The multiple regression method was used to analyze the significant predictors of the variable "Saying positive things about the pilgrimage". See Table 6.

**Table 6:** Motivations and saying positive things (significant predictors).

Variable	Beta (standardized)	t	Sig.	Tolerance
Tourism and Escape	-0.124	-2.663	0.008	1.000
Religious Experience	0.341	7.345	0.000	1.000
Belief Experience	0.270	5.807	0.000	1.000
Shopping	-0.031	-0.666	0.506	1.000
(Constant)		116.348	0.000	
F Test	23.799			
Sig.	0.000			
Durbin Watson	1.757			

According to the results from Table 6, the model was significant, without multicollinearity and autocorrelation. Three dimensions were significant predictors (p<0.05) for intentions to speak positively about religious events such as the pilgrimage. The dimension of religious experience, with a beta intensity of 0.341, positively correlated with speaking positively about the pilgrimage. Therefore, attendees with higher motivation levels for religious experience had greater intentions to speak positively about the pilgrimage. Similarly, the dimension of Belief Experience (beta = 0.270) positively correlates with speaking positively about the pilgrimage. Thus, more motivated attendees, by the belief experience, had greater intentions of speaking positively about the pilgrimage. In contrast, the Tourism and Escape dimension was negatively correlated with speaking positively about the pilgrimage. Therefore, more motivated attendees by tourism and escape had fewer intentions of speaking positively about the pilgrimage, which addresses our third research question. RQ3. What motivational factors predict loyalty in religious tourism focused on pilgrimages?

## 5. Discussion

The first objective of the research was to identify the main motivational factors of religious tourism focused on pilgrimages. This study found the factor "Tourism and Escape," also identified in other studies, such as Liro [31], which found tourist motivations. Another factor found in this study was "Religious Experience." Previous studies have found similar results, such as the religious motivation found by several authors like Lois-González & Santos, Bideci & Albayrak, Pillai et al., and Giușcă [24, 26, 27, 29]. Kamenidou & Vourou [25] identified pilgrimage motivations, and Piramanayagam et al. [30] identified motivations for religious belief. Another factor found in this study was motivations related to "Belief Experience." Similar studies have found similar results, such as the spiritual motivations found by several authors like Kamenidou & Vourou, Bideci & Albayrak, Thomas et al., Giușcă, and Kayal [20, 25, 26, 29, 34]. While Pillai et al. [27] found motivation for belief in experience, Almuhrzi & Alsawafi [28] found the search for faith. Another factor found in this study was the motivational dimension related to shopping. These findings have been identified in a few studies, such as Lois-González & Santos, Liro, and Hassan et al‥ [24, 31, 32]. The contribution of this study is that it has identified motivations that have been studied less in academic literature, such as motivations for tourism and escape and motivations for shopping. This indicates that tourists visit religious sites for spiritual and religious reasons and attend these events to engage in tourist activities and shopping.

Another objective was to analyze the motivational dimensions that predict satisfaction in religious tourism focused on pilgrimages. This study found that belief experience and religious experience were significant predictors of attendee satisfaction. Several studies have only found whether motivations influenced satisfaction, as is the case with Bond et al., Battour et al., Verma & Sarangi [14, 37, 39]. However, these previous studies did not identify the motivational factors influencing satisfaction. Meanwhile, Hassan et al. [32] found that religious experience influences satisfaction. This indicates that few studies aimed to identify significant motivational predictors of satisfaction. Therefore, this study contributes to the gap in the literature regarding predictors influencing satisfaction. Motivations for belief experience and religious experiences were the factors that influenced attendee satisfaction in the pilgrimage. On the other hand, tourist motivations and motivations for shopping did not influence satisfaction. These findings are essential for organizers of religious events to plan religious and belief activities that positively influence attendee satisfaction in the pilgrimage.

On the other hand, another objective of this study was to analyze the motivational dimensions that predict loyalty in religious tourism focused on pilgrimages. This study found that religious experience and belief experience were the motivational predictors that positively influenced attendees’ loyalty to the pilgrimage. Several studies have found a positive influence between motivations and loyalty, as seen in the studies by Siregar et al., Verma & Sarangi, Huang et al., Kamath et al. [14, 46, 47, 51]. Meanwhile, Hassan et al. [50] found that the religious factor would be the primary motivation for loyalty. So, it indicates that previous studies only confirmed the influence of motivations on loyalty but did not study which factors influenced loyalty. This study contributes to the literature by finding that religious experience and belief experience are the motivational factors that influence loyalty. These findings are essential for planning religious events to enhance activities related to attendees’ religious and belief experiences, allowing for increased loyalty among attendees to these religious events.Among the theoretical implications, it is noted that belief experience and religious experience influence the increase in satisfaction and loyalty of attendees at religious events.

Regarding the practical implications, organizers of religious events, service providers, and vendors offering religious products at these events should contribute to enhancing attendees’ religious and belief experiences. They can be achieved by improving religious facilities, better organizing religious activities, enhancing services related to these religious events, and improving the quality of religious products offered. In this way, the religious and belief experience can be enhanced, allowing for increased levels of satisfaction and loyalty towards attending the pilgrimage.

## 6. Conclusions

Religious motivations are essential in planning religious events such as pilgrimages, as they lead attendees to travel for various reasons, including religious experience, belief, tourism, escapism, and shopping. Not only is the study of motivations important in these events, but understanding the motivational dimensions that influence essential variables such as satisfaction and loyalty of attendees to pilgrimages is also crucial. In this sense, certain motivations that influence the satisfaction and loyalty of attendees to religious events like pilgrimages have been identified. Among them, the motivational dimension for religious experience includes appreciation for the architecture and beauty of the sanctuary and the motivation to attend the religious festival. Another dimension is the belief in experiencing a holy atmosphere, respecting the Virgin’s relics, and seeking redemption/liberation from suffering, which implies that those responsible for planning religious events and service providers should take a greater interest in knowing about the motivations that influence the satisfaction and loyalty of attendees to pilgrimages.

The religious and belief experience, facilities, services, and products related to religious events such as pilgrimages should be enhanced to improve. Meanwhile, return visits, recommendations, and positive feedback from attendees of religious events. Additionally, there will be benefits to businesses and services related to religious events, as there will be increased return visits and recommendations from attendees of religious events like pilgrimages.

Limitations of this study include that the sample was conducted in a specific pilgrimage; therefore, it may vary in other religious events. As a future line of research, analyzing the segmentation of attendees at religious events could be explored.

## Supporting information

S1 Checklist Human participants research checklist. (DOCX)

S1 Data (XLSX)
